# Benzyl 3-[(*E*)-2-nitro­benzyl­idene]dithio­carbazate

**DOI:** 10.1107/S1600536811028686

**Published:** 2011-07-23

**Authors:** Shang Shan, Yan-Lan Huang, Han-Qi Guo, Deng-Feng Li, Jian Sun

**Affiliations:** a College of Chemical Engineering and Materials Science, Zhejiang University of Technology, People’s Republic of China

## Abstract

The title compound, C_15_H_13_N_3_O_2_S_2_, was obtained from a condensation reaction of benzyl dithio­carbazate and 2-nitro­benzaldehyde. In the mol­ecule, the nearly planar dithio­carbazate fragment [r.m.s deviation = 0.0264 Å] is oriented at dihedral angles of 7.25 (17) and 74.09 (9)°with respect to the two benzene rings. The nitro group is twisted by a dihedral angle of 22.4 (7)° to the attached benzene ring. The nitro­benzene ring and dithio­carbazate fragment are located on the opposite sides of the C=N bond, showing an *E* configuration. In the crystal, mol­ecules are linked *via* inter­molecular N—H⋯S hydrogen bonds, forming centrosymmetric supra­molecular dimers. Weak C—H⋯π inter­action is also observed in the crystal structure.

## Related literature

For applications of hydrazone and its derivatives in the biological field, see: Okabe *et al.* (1993 ▶); Hu *et al.* (2001 ▶). For related structures, see: Shan *et al.* (2006 ▶, 2008*a* ▶,*b* ▶, 2011 ▶). For the synthesis, see: Hu *et al.* (2001 ▶).
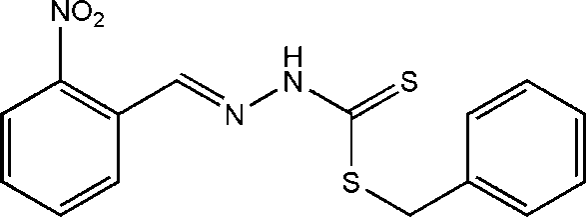


## Experimental

### 

#### Crystal data

C_15_H_13_N_3_O_2_S_2_
*M* *_r_* = 331.40
Monoclinic, 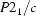

*a* = 4.673 (2) Å
*b* = 28.498 (6) Å
*c* = 11.735 (5) Å
β = 94.070 (4)°
*V* = 1558.8 (10) Å^3^
*Z* = 4
Mo *K*α radiation
μ = 0.35 mm^−1^
*T* = 294 K
0.38 × 0.25 × 0.23 mm

#### Data collection

Rigaku R-AXIS RAPID IP diffractometer
Absorption correction: multi-scan (*ABSCOR*; Higashi, 1995 ▶) *T*_min_ = 0.87, *T*_max_ = 0.94
6957 measured reflections
2814 independent reflections
1925 reflections with *I* > 2σ(*I*)
*R*_int_ = 0.041

#### Refinement

*R*[*F* ^2^ > 2σ(*F* ^2^)] = 0.055
*wR*(*F* ^2^) = 0.147
*S* = 1.05
2814 reflections
199 parameters
H-atom parameters constrained
Δρ_max_ = 0.63 e Å^−3^
Δρ_min_ = −0.47 e Å^−3^

### 

Data collection: *PROCESS-AUTO* (Rigaku, 1998 ▶); cell refinement: *PROCESS-AUTO*; data reduction: *CrystalStructure* (Rigaku/MSC, 2002 ▶); program(s) used to solve structure: *SIR92* (Altomare *et al.*, 1993 ▶); program(s) used to refine structure: *SHELXL97* (Sheldrick, 2008 ▶); molecular graphics: *ORTEP-3 for Windows* (Farrugia, 1997 ▶); software used to prepare material for publication: *WinGX* (Farrugia, 1999 ▶).

## Supplementary Material

Crystal structure: contains datablock(s) I, global. DOI: 10.1107/S1600536811028686/xu5268sup1.cif

Structure factors: contains datablock(s) I. DOI: 10.1107/S1600536811028686/xu5268Isup2.hkl

Supplementary material file. DOI: 10.1107/S1600536811028686/xu5268Isup3.cml

Additional supplementary materials:  crystallographic information; 3D view; checkCIF report

## Figures and Tables

**Table 1:** Hydrogen-bond geometry (Å, °) *Cg* is the centroid of the C10–C15 ring.

*D*—H⋯*A*	*D*—H	H⋯*A*	*D*⋯*A*	*D*—H⋯*A*
N2—H2⋯S1^i^	0.86	2.51	3.359 (3)	171
C9—H9*B*⋯*Cg*^ii^	0.97	2.50	3.410 (4)	156

Symmetry codes: (i) 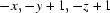; (ii) 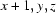.

**Fig. 1.**
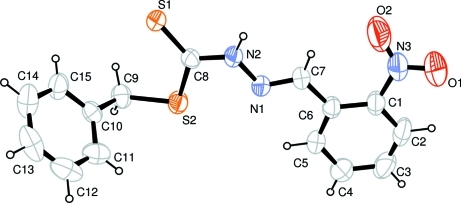
The molecular structure of the title compound with 40% probability displacement (arbitrary spheres for H atoms).

## supplementary crystallographic information

### Comment

Hydrazone and its derivatives have shown the potential application in the biological field (Okabe *et al.*, 1993; Hu *et al.*, 2001). As part of the ongoing investigation on anti-cancer compounds, the title compound has recently been prepared in our laboratory and its crystal structure is presented here.

The molecular structure of the title compound is shown in Fig. 1. In the molecule the nearly planar dithiocarbazate fragment [r.m.s deviation 0.0264 Å] is oriented with respect to the two benzene rings at 7.25 (17) and 74.09 (9)°, respectively. The nitro group is twisted to the attached-benzene ring with a dihedral angle of 22.4 (7)°. The N1—C7 bond length of 1.272 (4) Å indicates a typical C═N double bonds. The nitrobenzene ring and dithiocarbazate fragment are located on the opposite positions of the C═N bonds, showing the E-configuration, which agrees with those found in related compounds (Shan *et al.*, 2006; Shan *et al.*, 2008*a*,*b*); Shan *et al.* 2011). In the crystal the molecules are linked to each other via intermolecular N—H···S hydrogen bonding to form the centro-symmetric supramolecular dimer (Table 1). Weak C—H···π interaction is also observed in the crystal structure.

### Experimental

Benzyl dithiocarbazate was synthesized as described previously (Hu *et al.*, 2001). Benzyl dithiocarbazate (0.4 g, 2 mmol) and 2-nitrobenzaldehyde (0.3 g, 2 mmol) were dissolved in ethanol (20 ml), then acetic acid (0.2 ml) was added to the ethanol solution with stirring. The mixture solution was refluxed for 6 h. After cooling to room temperature, yellow microcrystals appeared. The microcrystals were separated from the solution and washed with cold water three times. Recrystallization was performed twice with absolute methanol to obtain single crystals of the title compound.

### Refinement

H atoms were placed in calculated positions with C—H = 0.93 (aromatic), 0.97 (methylene) and N—H = 0.86 Å, and refined in riding mode with *U*_iso_(H) = 1.2*U*_eq_(C,*N*).

### Crystal data

**Table:**

C_15_H_13_N_3_O_2_S_2_	*F*(000) = 688
*M**_r_* = 331.40	*D*_x_ = 1.412 Mg m^−^^3^
Monoclinic, *P*2_1_/*c*	Mo *K*α radiation, λ = 0.71073 Å
Hall symbol: -P 2ybc	Cell parameters from 2814 reflections
*a* = 4.673 (2) Å	θ = 2.7–25.2°
*b* = 28.498 (6) Å	µ = 0.35 mm^−^^1^
*c* = 11.735 (5) Å	*T* = 294 K
β = 94.070 (4)°	Needle, yellow
*V* = 1558.8 (10) Å^3^	0.38 × 0.25 × 0.23 mm
*Z* = 4	

### Data collection

**Table:**

Rigaku R-AXIS RAPID IP diffractometer	2814 independent reflections
Radiation source: fine-focus sealed tube	1925 reflections with *I* > 2σ(*I*)
graphite	*R*_int_ = 0.041
Detector resolution: 10.0 pixels mm^-1^	θ_max_ = 25.2°, θ_min_ = 2.8°
ω scans	*h* = −5→5
Absorption correction: multi-scan (*ABSCOR*; Higashi, 1995)	*k* = −34→33
*T*_min_ = 0.87, *T*_max_ = 0.94	*l* = −10→14
6957 measured reflections	

### Refinement

**Table:**

Refinement on *F*^2^	Primary atom site location: structure-invariant direct methods
Least-squares matrix: full	Secondary atom site location: difference Fourier map
*R*[*F*^2^ > 2σ(*F*^2^)] = 0.055	Hydrogen site location: inferred from neighbouring sites
*wR*(*F*^2^) = 0.147	H-atom parameters constrained
*S* = 1.05	*w* = 1/[σ^2^(*F*_o_^2^) + (0.0677*P*)^2^ + 0.3832*P*] where *P* = (*F*_o_^2^ + 2*F*_c_^2^)/3
2814 reflections	(Δ/σ)_max_ = 0.001
199 parameters	Δρ_max_ = 0.63 e Å^−^^3^
0 restraints	Δρ_min_ = −0.47 e Å^−^^3^

### Special details

**Table:**

Geometry. All e.s.d.'s (except the e.s.d. in the dihedral angle between two l.s. planes) are estimated using the full covariance matrix. The cell e.s.d.'s are taken into account individually in the estimation of e.s.d.'s in distances, angles and torsion angles; correlations between e.s.d.'s in cell parameters are only used when they are defined by crystal symmetry. An approximate (isotropic) treatment of cell e.s.d.'s is used for estimating e.s.d.'s involving l.s. planes.
Refinement. Refinement of *F*^2^ against ALL reflections. The weighted *R*-factor *wR* and goodness of fit *S* are based on *F*^2^, conventional *R*-factors *R* are based on *F*, with *F* set to zero for negative *F*^2^. The threshold expression of *F*^2^ > σ(*F*^2^) is used only for calculating *R*-factors(gt) *etc*. and is not relevant to the choice of reflections for refinement. *R*-factors based on *F*^2^ are statistically about twice as large as those based on *F*, and *R*- factors based on ALL data will be even larger.

### Fractional atomic coordinates and isotropic or equivalent isotropic displacement parameters (Å^2^)

**Table:**

	*x*	*y*	*z*	*U*_iso_*/*U*_eq_	
S1	0.1945 (2)	0.55533 (3)	0.40002 (8)	0.0502 (3)	
S2	0.53350 (19)	0.50159 (3)	0.23425 (8)	0.0520 (3)	
N1	0.3569 (6)	0.42391 (8)	0.3495 (2)	0.0448 (7)	
N2	0.2412 (6)	0.46411 (8)	0.3892 (2)	0.0462 (7)	
H2	0.1235	0.4627	0.4423	0.055*	
N3	0.2496 (8)	0.29447 (12)	0.5248 (3)	0.0760 (10)	
O1	0.1632 (9)	0.25714 (11)	0.5505 (3)	0.1173 (13)	
O2	0.2227 (15)	0.32692 (14)	0.5852 (5)	0.201 (3)	
C1	0.4074 (7)	0.29939 (11)	0.4221 (3)	0.0513 (9)	
C2	0.5231 (9)	0.25869 (11)	0.3821 (4)	0.0663 (11)	
H2A	0.4958	0.2303	0.4190	0.080*	
C3	0.6797 (9)	0.26018 (13)	0.2870 (4)	0.0725 (12)	
H3	0.7596	0.2329	0.2595	0.087*	
C4	0.7165 (8)	0.30232 (13)	0.2332 (4)	0.0659 (11)	
H4	0.8221	0.3036	0.1691	0.079*	
C5	0.5979 (8)	0.34250 (11)	0.2739 (3)	0.0539 (9)	
H5	0.6260	0.3707	0.2364	0.065*	
C6	0.4374 (7)	0.34259 (10)	0.3691 (3)	0.0452 (8)	
C7	0.3121 (7)	0.38673 (10)	0.4055 (3)	0.0495 (9)	
H7	0.2018	0.3876	0.4684	0.059*	
C8	0.3103 (7)	0.50609 (10)	0.3458 (3)	0.0406 (7)	
C9	0.5817 (7)	0.56211 (11)	0.1951 (3)	0.0499 (9)	
H9A	0.6092	0.5805	0.2646	0.060*	
H9B	0.7566	0.5645	0.1556	0.060*	
C10	0.3412 (6)	0.58388 (11)	0.1207 (3)	0.0422 (8)	
C11	0.2337 (8)	0.56274 (14)	0.0219 (3)	0.0620 (10)	
H11	0.3021	0.5334	0.0021	0.074*	
C12	0.0259 (9)	0.58440 (18)	−0.0482 (4)	0.0754 (12)	
H12	−0.0452	0.5698	−0.1151	0.091*	
C13	−0.0762 (9)	0.62724 (17)	−0.0198 (4)	0.0783 (14)	
H13	−0.2157	0.6419	−0.0677	0.094*	
C14	0.0259 (8)	0.64873 (13)	0.0791 (4)	0.0731 (13)	
H14	−0.0454	0.6778	0.0992	0.088*	
C15	0.2341 (7)	0.62698 (11)	0.1483 (4)	0.0560 (10)	
H15	0.3044	0.6417	0.2152	0.067*	

### Atomic displacement parameters (Å^2^)

**Table:**

	*U*^11^	*U*^22^	*U*^33^	*U*^12^	*U*^13^	*U*^23^
S1	0.0721 (6)	0.0349 (4)	0.0446 (5)	0.0042 (4)	0.0116 (4)	0.0001 (4)
S2	0.0618 (6)	0.0402 (4)	0.0561 (6)	0.0052 (4)	0.0197 (5)	0.0039 (4)
N1	0.0575 (16)	0.0352 (14)	0.0415 (16)	0.0028 (12)	0.0008 (14)	0.0020 (13)
N2	0.0601 (16)	0.0347 (14)	0.0451 (17)	0.0064 (12)	0.0132 (14)	0.0034 (13)
N3	0.107 (3)	0.0466 (19)	0.076 (3)	0.0025 (19)	0.019 (2)	0.0149 (19)
O1	0.170 (4)	0.076 (2)	0.110 (3)	−0.038 (2)	0.042 (3)	0.023 (2)
O2	0.406 (9)	0.068 (2)	0.152 (4)	0.020 (4)	0.178 (5)	0.029 (3)
C1	0.059 (2)	0.0388 (18)	0.055 (2)	−0.0062 (15)	−0.0015 (18)	0.0084 (17)
C2	0.080 (3)	0.0318 (17)	0.085 (3)	−0.0018 (17)	−0.009 (2)	0.008 (2)
C3	0.088 (3)	0.041 (2)	0.088 (3)	0.0135 (19)	0.006 (3)	−0.006 (2)
C4	0.081 (3)	0.055 (2)	0.063 (3)	0.0091 (19)	0.012 (2)	−0.002 (2)
C5	0.068 (2)	0.0364 (17)	0.057 (2)	0.0012 (16)	0.005 (2)	0.0052 (17)
C6	0.0516 (19)	0.0356 (16)	0.047 (2)	−0.0011 (14)	−0.0061 (17)	0.0048 (15)
C7	0.066 (2)	0.0368 (17)	0.046 (2)	−0.0010 (16)	0.0056 (18)	0.0076 (16)
C8	0.0456 (17)	0.0395 (16)	0.0358 (18)	0.0000 (13)	−0.0041 (15)	0.0037 (14)
C9	0.0454 (18)	0.0479 (18)	0.057 (2)	−0.0060 (15)	0.0100 (17)	0.0089 (17)
C10	0.0396 (17)	0.0466 (18)	0.041 (2)	−0.0072 (14)	0.0089 (15)	0.0093 (16)
C11	0.064 (2)	0.075 (2)	0.048 (2)	−0.001 (2)	0.014 (2)	−0.007 (2)
C12	0.073 (3)	0.111 (4)	0.041 (2)	−0.020 (3)	−0.001 (2)	0.003 (2)
C13	0.057 (2)	0.090 (3)	0.085 (4)	−0.010 (2)	−0.009 (2)	0.048 (3)
C14	0.067 (3)	0.047 (2)	0.103 (4)	−0.0021 (19)	−0.009 (3)	0.018 (2)
C15	0.060 (2)	0.0395 (18)	0.067 (3)	−0.0069 (16)	−0.006 (2)	0.0060 (18)

### Geometric parameters (Å, °)

**Table:**

S1—C8	1.648 (3)	C5—C6	1.389 (5)
S2—C8	1.735 (3)	C5—H5	0.9300
S2—C9	1.803 (3)	C6—C7	1.464 (4)
N1—C7	1.272 (4)	C7—H7	0.9300
N1—N2	1.363 (3)	C9—C10	1.507 (5)
N2—C8	1.349 (4)	C9—H9A	0.9700
N2—H2	0.8600	C9—H9B	0.9700
N3—O2	1.178 (5)	C10—C11	1.370 (5)
N3—O1	1.185 (4)	C10—C15	1.374 (5)
N3—C1	1.464 (5)	C11—C12	1.374 (6)
C1—C2	1.376 (5)	C11—H11	0.9300
C1—C6	1.390 (4)	C12—C13	1.361 (6)
C2—C3	1.377 (6)	C12—H12	0.9300
C2—H2A	0.9300	C13—C14	1.368 (6)
C3—C4	1.373 (5)	C13—H13	0.9300
C3—H3	0.9300	C14—C15	1.370 (5)
C4—C5	1.373 (5)	C14—H14	0.9300
C4—H4	0.9300	C15—H15	0.9300
			
C8—S2—C9	102.38 (15)	C6—C7—H7	120.6
C7—N1—N2	116.1 (3)	N2—C8—S1	121.0 (2)
C8—N2—N1	120.3 (3)	N2—C8—S2	113.1 (2)
C8—N2—H2	119.8	S1—C8—S2	125.83 (18)
N1—N2—H2	119.8	C10—C9—S2	116.1 (2)
O2—N3—O1	119.9 (4)	C10—C9—H9A	108.3
O2—N3—C1	120.2 (4)	S2—C9—H9A	108.3
O1—N3—C1	119.8 (4)	C10—C9—H9B	108.3
C2—C1—C6	122.6 (4)	S2—C9—H9B	108.3
C2—C1—N3	115.7 (3)	H9A—C9—H9B	107.4
C6—C1—N3	121.7 (3)	C11—C10—C15	118.3 (3)
C1—C2—C3	119.6 (4)	C11—C10—C9	121.5 (3)
C1—C2—H2A	120.2	C15—C10—C9	120.1 (3)
C3—C2—H2A	120.2	C10—C11—C12	120.7 (4)
C4—C3—C2	119.4 (4)	C10—C11—H11	119.7
C4—C3—H3	120.3	C12—C11—H11	119.7
C2—C3—H3	120.3	C13—C12—C11	120.1 (4)
C5—C4—C3	120.2 (4)	C13—C12—H12	119.9
C5—C4—H4	119.9	C11—C12—H12	119.9
C3—C4—H4	119.9	C12—C13—C14	120.2 (4)
C4—C5—C6	122.4 (3)	C12—C13—H13	119.9
C4—C5—H5	118.8	C14—C13—H13	119.9
C6—C5—H5	118.8	C13—C14—C15	119.4 (4)
C5—C6—C1	115.8 (3)	C13—C14—H14	120.3
C5—C6—C7	119.1 (3)	C15—C14—H14	120.3
C1—C6—C7	125.1 (3)	C14—C15—C10	121.3 (4)
N1—C7—C6	118.8 (3)	C14—C15—H15	119.3
N1—C7—H7	120.6	C10—C15—H15	119.3

### Hydrogen-bond geometry (Å, °)

**Table:**

Cg is the centroid of the C10–C15 ring.

**Table:**

*D*—H···*A*	*D*—H	H···*A*	*D*···*A*	*D*—H···*A*
N2—H2···S1^i^	0.86	2.51	3.359 (3)	171
C9—H9B···Cg^ii^	0.97	2.50	3.410 (4)	156

Symmetry codes: (i) −*x*, −*y*+1, −*z*+1; (ii) *x*+1, *y*, *z*.
